# Investigating the contribution of subcortical salience network structures to a measure of social cognition across neurodegenerative diseases

**DOI:** 10.1002/alz.088546

**Published:** 2025-01-09

**Authors:** Carly Davenport, Mohsen Hadian, Indira Ruth Garcia Cordero, Andrew R Frank, Anthony E Lang, Angela C Roberts, Angela Troyer, Brian Levine, Brian Tan, Corinne E. Fischer, Connie Marras, Donna Kwan, David F. Tang‐Wai, Elizabeth Finger, Joseph B Orange, Joel Ramirez, Sean Symons, Kelly M Sunderland, Michael Borrie, Mario Masellis, Douglas P Munoz, Richard H Swartz, Morris Freedman, Miracle Ozzoude, Paula M McLaughlin, Robert Bartha, Michael J. Strong, Maria Carmela Tartaglia, the ONDRI Investigators

**Affiliations:** ^1^ Tanz Centre for Research in Neurodegenerative Diseases, University of Toronto, Toronto, ON Canada; ^2^ Bruyere Research Institute, Ottawa, ON Canada; ^3^ The Edmond J. Safra Program in Parkinson’s Disease and Morton and Gloria Shulman Movement Disorders Clinic, Toronto, ON Canada; ^4^ Western University, London, ON Canada; ^5^ Baycrest Health Sciences Centre, Toronto, ON Canada; ^6^ Rotman Research Institute, Toronto, ON Canada; ^7^ Rotman Research Institute, Baycrest Health Sciences, Toronto, ON Canada; ^8^ Keenan Research Centre for Biomedical Science, Li Ka Shing Knowledge Institute, St. Michael's Hospital, Toronto, ON Canada; ^9^ Krembil Research Institute, University Health Network, Toronto, ON Canada; ^10^ Queens University, Kingston, ON Canada; ^11^ Memory Clinic, Toronto Western Hospital, University Health Network, Toronto, ON Canada; ^12^ Department of Clinical Neurological Sciences, University of Western Ontario, London, ON Canada; ^13^ Sunnybrook Health Sciences Centre, Toronto, ON Canada; ^14^ Rotman Research Institute, Baycrest Health Sciences Centre, Toronto, ON Canada; ^15^ Division of Geriatric Medicine, Western University, London, ON Canada; ^16^ Queen's University, Kingston, ON Canada; ^17^ Nova Scotia Health, Halifax, NS Canada; ^18^ Department of Clinical Neurological Sciences and The Robarts Research Institute, Western University, London, ON Canada

## Abstract

**Background:**

Social cognition is impacted early in the disease progression of many neurodegenerative diseases (ND). The Salience network (SN) is an intrinsically connected brain network responsible for social cognitive function. Keys hubs of this brain network, the anterior insula (AI) and anterior cingulate cortex (ACC), are reported to incorporate ‘bottom‐up’ signals from subcortical regions such as the amygdala and periaqueductal gray (PAG), but this mechanism and the subcortical contribution to SN connectivity is poorly understood. Our aim was to investigate the contribution of cortical and subcortical structures to SN functional connectivity and to social cognition across NDs.

**Method:**

76 participants (21 Alzheimer’s disease, 13 behavioural variant frontotemporal dementia, and 42 Parkinson’s disease) from the Ontario Neurodegenerative Research Initiative (ONDRI) baseline or one‐year follow up visits with resting state fMRI, Montreal Cognitive Assessment (MoCA) total scores, and informant‐reported socioemotional sensitivity scores using the Revised Self‐Monitoring Scale (RSMS) were included (higher score, indicating higher function). All groups were age‐ and sex‐matched. Fisher‐transformed correlation coefficients of functional connectivity from an ROI‐to‐ROI analysis between cortical and subcortical SN ROIs were used to create a mean cortical SN value and mean subcortical SN value to use in linear regression modelling with behavioural scores.

**Result:**

Mean cortical and subcortical SN connectivity were significantly associated with RSMS total score (b = 2.94, p = 0.041; (b = 3.60, p = 0.014, respectively), independent of cognitive function, with higher connectivity predicting higher score. The interaction between cortical and subcortical connectivity was not significantly associated with RSMS total score. Mean cortical and subcortical connectivity was significantly associated with RSMS‐EX (expressive behaviour of others) and RSMS‐SP (self‐presentation) subscores (b = 1.36, p = 0.049; b = 1.44, p = 0.040; b coef = 1.58, p‐value = 0.033; b coef = 2.15, p‐value = 0.005, respectively).

**Conclusion:**

Our results indicate a stronger contribution of subcortical structures to social cognition‐related functional connectivity across various neurodegenerative diseases. Despite previous associations with cortical regions, our evidence suggests that alterations in subcortical structures mediate changes in social cognition. Further exploration in larger cohorts is necessary, as impaired social cognition in patients with ND is associated with increased caregiver distress.

